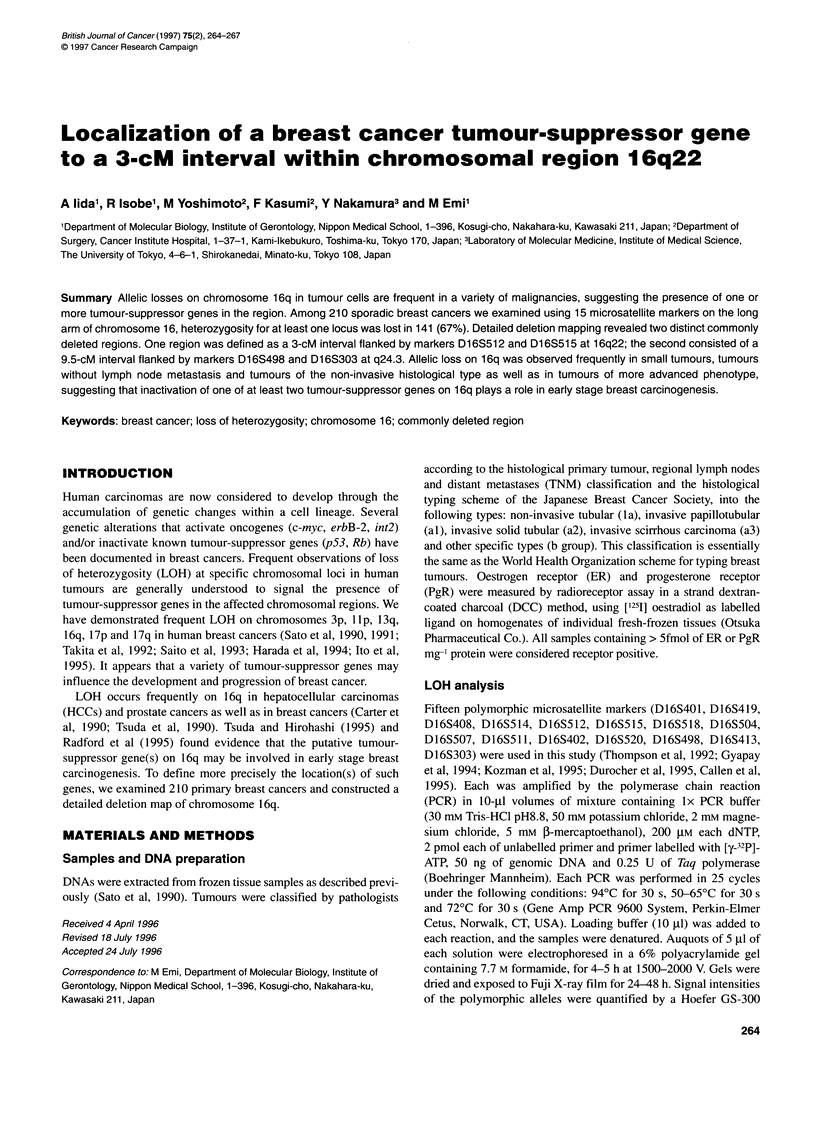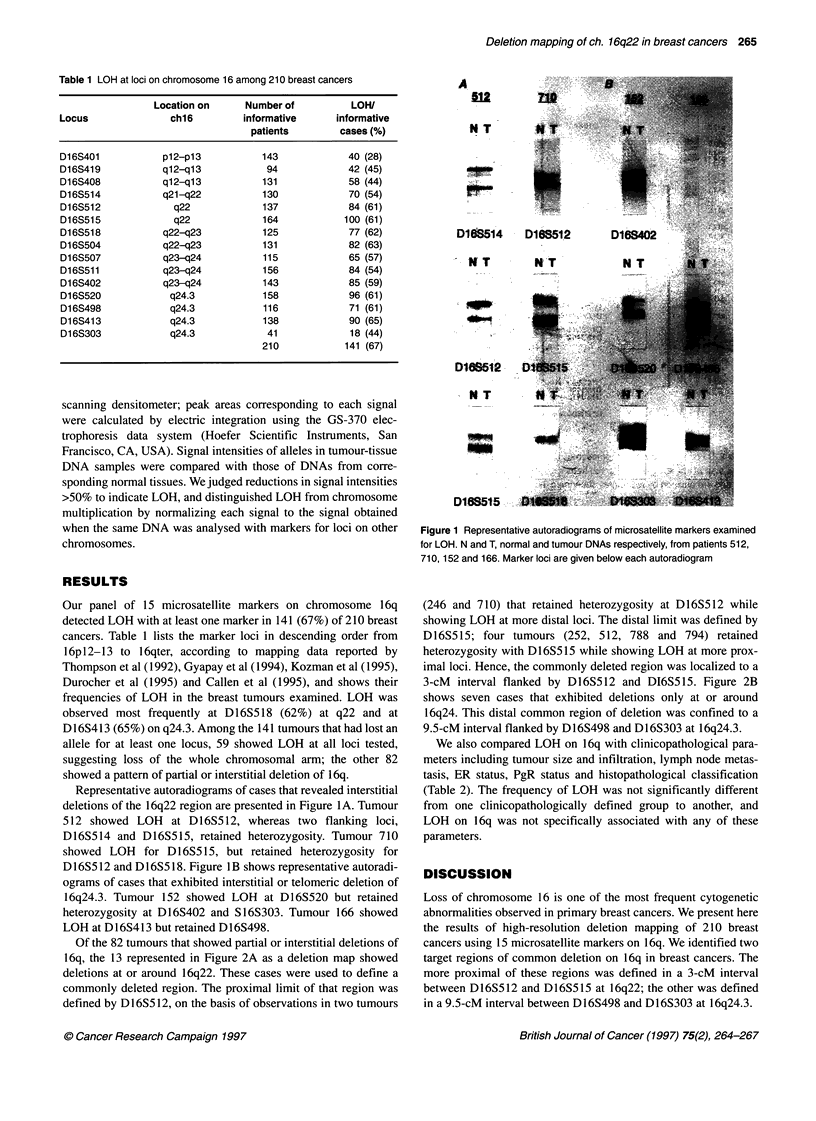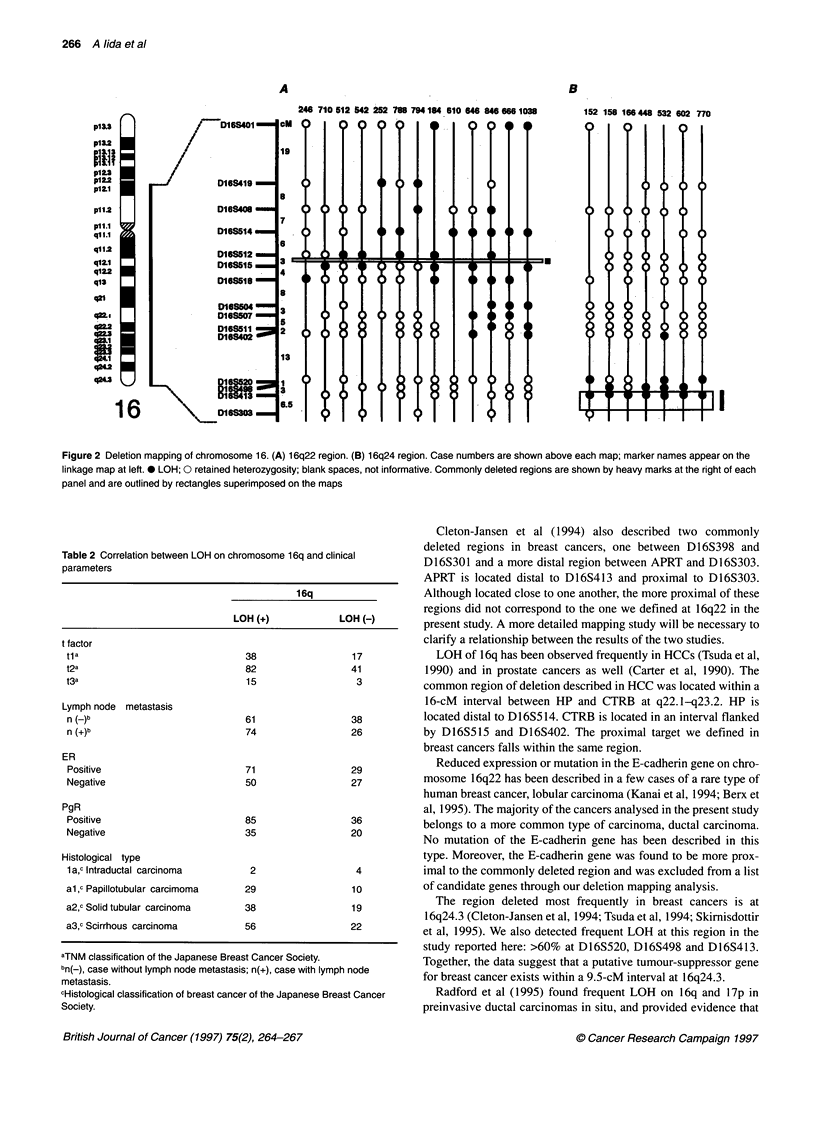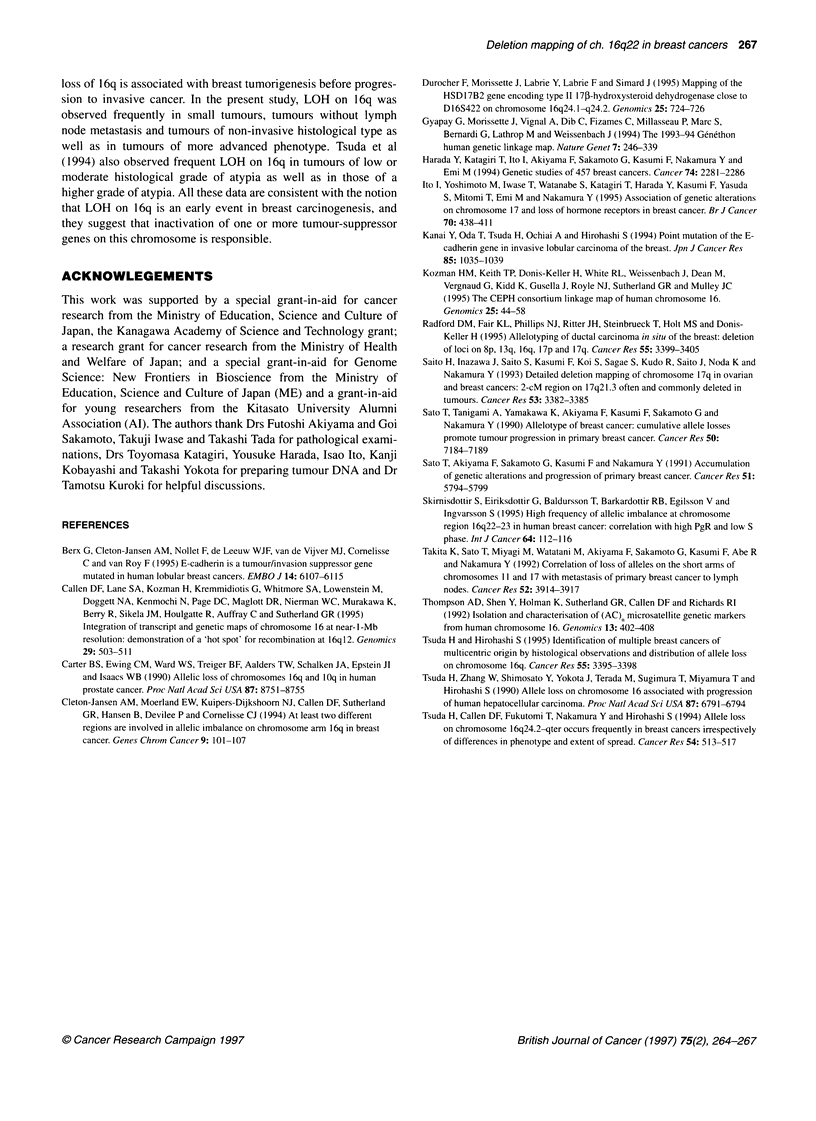# Localization of a breast cancer tumour-suppressor gene to a 3-cM interval within chromosomal region 16q22.

**DOI:** 10.1038/bjc.1997.43

**Published:** 1997

**Authors:** A. Iida, R. Isobe, M. Yoshimoto, F. Kasumi, Y. Nakamura, M. Emi

**Affiliations:** Department of Molecular Biology, Nippon Medical School, Kawasaki, Japan.

## Abstract

**Images:**


British Journal of Cancer (1997) 75(2), 264–267
© 1997 Cancer Research Campaign

### Localization of a breast cancer tumour-suppressor gene to a 3-cM interval within chromosomal region 16q22

A Iida[1], R Isobe[1], M Yoshimoto[2], F Kasumi[2], Y Nakamura[3] and M Emi[1]

[1]Department of Molecular Biology, Institute of Gerontology, Nippon Medical School, 1–396, Kosugi-cho, Nakahara-ku, Kawasaki 211, Japan; [2]Department of Surgery, Cancer Institute Hospital, 1–37–1, Kami-Ikebukuro, Toshima-ku, Tokyo 170, Japan; [3]Laboratory of Molecular Medicine, Institute of Medical Science, The University of Tokyo, 4–6–1, Shirokanedai, Minato-ku, Tokyo 108, Japan

**Summary** Allelic losses on chromosome 16q in tumour cells are frequent in a variety of malignancies, suggesting the presence of one or more tumour-suppressor genes in the region. Among 210 sporadic breast cancers we examined using 15 microsatellite markers on the long arm of chromosome 16, heterozygosity for at least one locus was lost in 141 (67%). Detailed deletion mapping revealed two distinct commonly deleted regions. One region was defined as a 3-cM interval flanked by markers D16S512 and D16S515 at 16q22; the second consisted of a 9.5-cM interval flanked by markers D16S498 and D16S303 at q24.3. Allelic loss on 16q was observed frequently in small tumours, tumours without lymph node metastasis and tumours of the non-invasive histological type as well as in tumours of more advanced phenotype, suggesting that inactivation of one of at least two tumour-suppressor genes on 16q plays a role in early stage breast carcinogenesis.

**Keywords**: breast cancer; loss of heterozygosity; chromosome 16; commonly deleted region

### INTRODUCTION

Human carcinomas are now considered to develop through the accumulation of genetic changes within a cell lineage. Several genetic alterations that activate oncogenes (c-*myc*, *erb*B-2, *int2*) and/or inactivate known tumour-suppressor genes (*p53*, *Rb*) have been documented in breast cancers. Frequent observations of loss of heterozygosity (LOH) at specific chromosomal loci in human tumours are generally understood to signal the presence of tumour-suppressor genes in the affected chromosomal regions. We have demonstrated frequent LOH on chromosomes 3p, 11p, 13q, 16q, 17p and 17q in human breast cancers (Sato et al, 1990, 1991; Takita et al, 1992; Saito et al, 1993; Harada et al, 1994; Ito et al, 1995). It appears that a variety of tumour-suppressor genes may influence the development and progression of breast cancer.

LOH occurs frequently on 16q in hepatocellular carcinomas (HCCs) and prostate cancers as well as in breast cancers (Carter et al, 1990; Tsuda et al, 1990). Tsuda and Hirohashi (1995) and Radford et al (1995) found evidence that the putative tumour-suppressor gene(s) on 16q may be involved in early stage breast carcinogenesis. To define more precisely the location(s) of such genes, we examined 210 primary breast cancers and constructed a detailed deletion map of chromosome 16q.

### MATERIALS AND METHODS

### Samples and DNA preparation

DNAs were extracted from frozen tissue samples as described previously (Sato et al, 1990). Tumours were classified by pathologists according to the histological primary tumour, regional lymph nodes and distant metastases (TNM) classification and the histological typing scheme of the Japanese Breast Cancer Society, into the following types: non-invasive tubular (1a), invasive papillotubular (a1), invasive solid tubular (a2), invasive scirrhous carcinoma (a3) and other specific types (b group). This classification is essentially the same as the World Health Organization scheme for typing breast tumours. Oestrogen receptor (ER) and progesterone receptor (PgR) were measured by radioreceptor assay in a strand dextran-coated charcoal (DCC) method, using [$^{125}$I] oestradiol as labelled ligand on homogenates of individual fresh-frozen tissues (Otsuka Pharmaceutical Co.). All samples containing > 5fmol of ER or PgR $mg^{-1}$ protein were considered receptor positive.

### LOH analysis

Fifteen polymorphic microsatellite markers (D16S401, D16S419, D16S408, D16S514, D16S512, D16S515, D16S518, D16S504, D16S507, D16S511, D16S402, D16S520, D16S498, D16S413, D16S303) were used in this study (Thompson et al, 1992; Gyapay et al, 1994; Kozman et al, 1995; Durocher et al, 1995, Callen et al, 1995). Each was amplified by the polymerase chain reaction (PCR) in 10-µl volumes of mixture containing 1× PCR buffer (30 mM Tris-HCl pH8.8, 50 mM potassium chloride, 2 mM magnesium chloride, 5 mM β-mercaptoethanol), 200 µM each dNTP, 2 pmol each of unlabelled primer and primer labelled with [γ-$^{32}$P]-ATP, 50 ng of genomic DNA and 0.25 U of *Taq* polymerase (Boehringer Mannheim). Each PCR was performed in 25 cycles under the following conditions: 94°C for 30 s, 50–65°C for 30 s and 72°C for 30 s (Gene Amp PCR 9600 System, Perkin-Elmer Cetus, Norwalk, CT, USA). Loading buffer (10 µl) was added to each reaction, and the samples were denatured. Auquots of 5 µl of each solution were electrophoresed in a 6% polyacrylamide gel containing 7.7 M formamide, for 4–5 h at 1500–2000 V. Gels were dried and exposed to Fuji X-ray film for 24–48 h. Signal intensities of the polymorphic alleles were quantified by a Hoefer GS-300

*Received 4 April 1996*
*Revised 18 July 1996*
*Accepted 24 July 1996*

*Correspondence to:* M Emi, Department of Molecular Biology, Institute of Gerontology, Nippon Medical School, 1-396, Kosugi-cho, Nakahara-ku, Kawasaki 211, Japan

**Table 1** LOH at loci on chromosome 16 among 210 breast cancers

| Locus | Location on ch16 | Number of informative patients | LOH/ informative cases (%) |
|---|---|---|---|
| D16S401 | p12–p13 | 143 | 40 (28) |
| D16S419 | q12–q13 | 94 | 42 (45) |
| D16S408 | q12–q13 | 131 | 58 (44) |
| D16S514 | q21–q22 | 130 | 70 (54) |
| D16S512 | q22 | 137 | 84 (61) |
| D16S515 | q22 | 164 | 100 (61) |
| D16S518 | q22–q23 | 125 | 77 (62) |
| D16S504 | q22–q23 | 131 | 82 (63) |
| D16S507 | q23–q24 | 115 | 65 (57) |
| D16S511 | q23–q24 | 156 | 84 (54) |
| D16S402 | q23–q24 | 143 | 85 (59) |
| D16S520 | q24.3 | 158 | 96 (61) |
| D16S498 | q24.3 | 116 | 71 (61) |
| D16S413 | q24.3 | 138 | 90 (65) |
| D16S303 | q24.3 | 41 | 18 (44) |
| | | 210 | 141 (67) |

scanning densitometer; peak areas corresponding to each signal were calculated by electric integration using the GS-370 electrophoresis data system (Hoefer Scientific Instruments, San Francisco, CA, USA). Signal intensities of alleles in tumour-tissue DNA samples were compared with those of DNAs from corresponding normal tissues. We judged reductions in signal intensities >50% to indicate LOH, and distinguished LOH from chromosome multiplication by normalizing each signal to the signal obtained when the same DNA was analysed with markers for loci on other chromosomes.

**Figure 1** Representative autoradiograms of microsatellite markers examined for LOH. N and T, normal and tumour DNAs respectively, from patients 512, 710, 152 and 166. Marker loci are given below each autoradiogram

### RESULTS

Our panel of 15 microsatellite markers on chromosome 16q detected LOH with at least one marker in 141 (67%) of 210 breast cancers. Table 1 lists the marker loci in descending order from 16p12–13 to 16qter, according to mapping data reported by Thompson et al (1992), Gyapay et al (1994), Kozman et al (1995), Durocher et al (1995) and Callen et al (1995), and shows their frequencies of LOH in the breast tumours examined. LOH was observed most frequently at D16S518 (62%) at q22 and at D16S413 (65%) on q24.3. Among the 141 tumours that had lost an allele for at least one locus, 59 showed LOH at all loci tested, suggesting loss of the whole chromosomal arm; the other 82 showed a pattern of partial or interstitial deletion of 16q.

Representative autoradiograms of cases that revealed interstitial deletions of the 16q22 region are presented in Figure 1A. Tumour 512 showed LOH at D16S512, whereas two flanking loci, D16S514 and D16S515, retained heterozygosity. Tumour 710 showed LOH for D16S515, but retained heterozygosity for D16S512 and D16S518. Figure 1B shows representative autoradiograms of cases that exhibited interstitial or telomeric deletion of 16q24.3. Tumour 152 showed LOH at D16S520 but retained heterozygosity at D16S402 and S16S303. Tumour 166 showed LOH at D16S413 but retained D16S498.

Of the 82 tumours that showed partial or interstitial deletions of 16q, the 13 represented in Figure 2A as a deletion map showed deletions at or around 16q22. These cases were used to define a commonly deleted region. The proximal limit of that region was defined by D16S512, on the basis of observations in two tumours (246 and 710) that retained heterozygosity at D16S512 while showing LOH at more distal loci. The distal limit was defined by D16S515; four tumours (252, 512, 788 and 794) retained heterozygosity with D16S515 while showing LOH at more proximal loci. Hence, the commonly deleted region was localized to a 3-cM interval flanked by D16S512 and DI6S515. Figure 2B shows seven cases that exhibited deletions only at or around 16q24. This distal common region of deletion was confined to a 9.5-cM interval flanked by D16S498 and D16S303 at 16q24.3.

We also compared LOH on 16q with clinicopathological parameters including tumour size and infiltration, lymph node metastasis, ER status, PgR status and histopathological classification (Table 2). The frequency of LOH was not significantly different from one clinicopathologically defined group to another, and LOH on 16q was not specifically associated with any of these parameters.

### DISCUSSION

Loss of chromosome 16 is one of the most frequent cytogenetic abnormalities observed in primary breast cancers. We present here the results of high-resolution deletion mapping of 210 breast cancers using 15 microsatellite markers on 16q. We identified two target regions of common deletion on 16q in breast cancers. The more proximal of these regions was defined in a 3-cM interval between D16S512 and D16S515 at 16q22; the other was defined in a 9.5-cM interval between D16S498 and D16S303 at 16q24.3.

© Cancer Research Campaign 1997

**Figure 2** Deletion mapping of chromosome 16. (**A**) 16q22 region. (**B**) 16q24 region. Case numbers are shown above each map; marker names appear on the linkage map at left. ● LOH; ○ retained heterozygosity; blank spaces, not informative. Commonly deleted regions are shown by heavy marks at the right of each panel and are outlined by rectangles superimposed on the maps

**Table 2** Correlation between LOH on chromosome 16q and clinical parameters

| | 16q | |
|---|---|---|
| | LOH (+) | LOH (–) |
| t factor | | |
| t1[a] | 38 | 17 |
| t2[a] | 82 | 41 |
| t3[a] | 15 | 3 |
| Lymph node metastasis | | |
| n (–)[b] | 61 | 38 |
| n (+)[b] | 74 | 26 |
| ER | | |
| Positive | 71 | 29 |
| Negative | 50 | 27 |
| PgR | | |
| Positive | 85 | 36 |
| Negative | 35 | 20 |
| Histological type | | |
| 1a,[c] Intraductal carcinoma | 2 | 4 |
| a1,[c] Papillotubular carcimoma | 29 | 10 |
| a2,[c] Solid tubular carcinoma | 38 | 19 |
| a3,[c] Scirrhous carcinoma | 56 | 22 |

[a]TNM classification of the Japanese Breast Cancer Society.
[b]n(–), case without lymph node metastasis; n(+), case with lymph node metastasis.
[c]Histological classification of breast cancer of the Japanese Breast Cancer Society.

Cleton-Jansen et al (1994) also described two commonly deleted regions in breast cancers, one between D16S398 and D16S301 and a more distal region between APRT and D16S303. APRT is located distal to D16S413 and proximal to D16S303. Although located close to one another, the more proximal of these regions did not correspond to the one we defined at 16q22 in the present study. A more detailed mapping study will be necessary to clarify a relationship between the results of the two studies.

LOH of 16q has been observed frequently in HCCs (Tsuda et al, 1990) and in prostate cancers as well (Carter et al, 1990). The common region of deletion described in HCC was located within a 16-cM interval between HP and CTRB at q22.1–q23.2. HP is located distal to D16S514. CTRB is located in an interval flanked by D16S515 and D16S402. The proximal target we defined in breast cancers falls within the same region.

Reduced expression or mutation in the E-cadherin gene on chromosome 16q22 has been described in a few cases of a rare type of human breast cancer, lobular carcinoma (Kanai et al, 1994; Berx et al, 1995). The majority of the cancers analysed in the present study belongs to a more common type of carcinoma, ductal carcinoma. No mutation of the E-cadherin gene has been described in this type. Moreover, the E-cadherin gene was found to be more proximal to the commonly deleted region and was excluded from a list of candidate genes through our deletion mapping analysis.

The region deleted most frequently in breast cancers is at 16q24.3 (Cleton-Jansen et al, 1994; Tsuda et al, 1994; Skirnisdottir et al, 1995). We also detected frequent LOH at this region in the study reported here: >60% at D16S520, D16S498 and D16S413. Together, the data suggest that a putative tumour-suppressor gene for breast cancer exists within a 9.5-cM interval at 16q24.3.

Radford et al (1995) found frequent LOH on 16q and 17p in preinvasive ductal carcinomas in situ, and provided evidence that

*© Cancer Research Campaign 1997*

loss of 16q is associated with breast tumorigenesis before progression to invasive cancer. In the present study, LOH on 16q was observed frequently in small tumours, tumours without lymph node metastasis and tumours of non-invasive histological type as well as in tumours of more advanced phenotype. Tsuda et al (1994) also observed frequent LOH on 16q in tumours of low or moderate histological grade of atypia as well as in those of a higher grade of atypia. All these data are consistent with the notion that LOH on 16q is an early event in breast carcinogenesis, and they suggest that inactivation of one or more tumour-suppressor genes on this chromosome is responsible.

### ACKNOWLEGEMENTS

This work was supported by a special grant-in-aid for cancer research from the Ministry of Education, Science and Culture of Japan, the Kanagawa Academy of Science and Technology grant; a research grant for cancer research from the Ministry of Health and Welfare of Japan; and a special grant-in-aid for Genome Science: New Frontiers in Bioscience from the Ministry of Education, Science and Culture of Japan (ME) and a grant-in-aid for young researchers from the Kitasato University Alumni Association (AI). The authors thank Drs Futoshi Akiyama and Goi Sakamoto, Takuji Iwase and Takashi Tada for pathological examinations, Drs Toyomasa Katagiri, Yousuke Harada, Isao Ito, Kanji Kobayashi and Takashi Yokota for preparing tumour DNA and Dr Tamotsu Kuroki for helpful discussions.

### REFERENCES

Berx G, Cleton-Jansen AM, Nollet F, de Leeuw WJF, van de Vijver MJ, Cornelisse C and van Roy F (1995) E-cadherin is a tumour/invasion suppressor gene mutated in human lobular breast cancers. *EMBO J* **14:** 6107–6115

Callen DF, Lane SA, Kozman H, Kremmidiotis G, Whitmore SA, Lowenstein M, Doggett NA, Kenmochi N, Page DC, Maglott DR, Nierman WC, Murakawa K, Berry R, Sikela JM, Houlgatte R, Auffray C and Sutherland GR (1995) Integration of transcript and genetic maps of chromosome 16 at near-1-Mb resolution: demonstration of a 'hot spot' for recombination at 16q12. *Genomics* **29:** 503–511

Carter BS, Ewing CM, Ward WS, Treiger BF, Aalders TW, Schalken JA, Epstein JI and Isaacs WB (1990) Allelic loss of chromosomes 16q and 10q in human prostate cancer. *Proc Natl Acad Sci USA* **87:** 8751–8755

Cleton-Jansen AM, Moerland EW, Kuipers-Dijkshoorn NJ, Callen DF, Sutherland GR, Hansen B, Devilee P and Cornelisse CJ (1994) At least two different regions are involved in allelic imbalance on chromosome arm 16q in breast cancer. *Genes Chrom Cancer* **9:** 101–107

Durocher F, Morissette J, Labrie Y, Labrie F and Simard J (1995) Mapping of the HSD17B2 gene encoding type II 17β-hydroxysteroid dehydrogenase close to D16S422 on chromosome 16q24.1–q24.2. *Genomics* **25:** 724–726

Gyapay G, Morissette J, Vignal A, Dib C, Fizames C, Millasseau P, Marc S, Bernardi G, Lathrop M and Weissenbach J (1994) The 1993–94 Généthon human genetic linkage map. *Nature Genet* **7:** 246–339

Harada Y, Katagiri T, Ito I, Akiyama F, Sakamoto G, Kasumi F, Nakamura Y and Emi M (1994) Genetic studies of 457 breast cancers. *Cancer* **74:** 2281–2286

Ito I, Yoshimoto M, Iwase T, Watanabe S, Katagiri T, Harada Y, Kasumi F, Yasuda S, Mitomi T, Emi M and Nakamura Y (1995) Association of genetic alterations on chromosome 17 and loss of hormone receptors in breast cancer. *Br J Cancer* **70:** 438–411

Kanai Y, Oda T, Tsuda H, Ochiai A and Hirohashi S (1994) Point mutation of the E-cadherin gene in invasive lobular carcinoma of the breast. *Jpn J Cancer Res* **85:** 1035–1039

Kozman HM, Keith TP, Donis-Keller H, White RL, Weissenbach J, Dean M, Vergnaud G, Kidd K, Gusella J, Royle NJ, Sutherland GR and Mulley JC (1995) The CEPH consortium linkage map of human chromosome 16. *Genomics* **25:** 44–58

Radford DM, Fair KL, Phillips NJ, Ritter JH, Steinbrueck T, Holt MS and Donis-Keller H (1995) Allelotyping of ductal carcinoma *in situ* of the breast: deletion of loci on 8p, 13q, 16q, 17p and 17q. *Cancer Res* **55:** 3399–3405

Saito H, Inazawa J, Saito S, Kasumi F, Koi S, Sagae S, Kudo R, Saito J, Noda K and Nakamura Y (1993) Detailed deletion mapping of chromosome 17q in ovarian and breast cancers: 2-cM region on 17q21.3 often and commonly deleted in tumours. *Cancer Res* **53:** 3382–3385

Sato T, Tanigami A, Yamakawa K, Akiyama F, Kasumi F, Sakamoto G and Nakamura Y (1990) Allelotype of breast cancer: cumulative allele losses promote tumour progression in primary breast cancer. *Cancer Res* **50:** 7184–7189

Sato T, Akiyama F, Sakamoto G, Kasumi F and Nakamura Y (1991) Accumulation of genetic alterations and progression of primary breast cancer. *Cancer Res* **51:** 5794–5799

Skirnisdottir S, Eiriksdottir G, Baldursson T, Barkardottir RB, Egilsson V and Ingvarsson S (1995) High frequency of allelic imbalance at chromosome region 16q22–23 in human breast cancer: correlation with high PgR and low S phase. *Int J Cancer* **64:** 112–116

Takita K, Sato T, Miyagi M, Watatani M, Akiyama F, Sakamoto G, Kasumi F, Abe R and Nakamura Y (1992) Correlation of loss of alleles on the short arms of chromosomes 11 and 17 with metastasis of primary breast cancer to lymph nodes. *Cancer Res* **52:** 3914–3917

Thompson AD, Shen Y, Holman K, Sutherland GR, Callen DF and Richards RI (1992) Isolation and characterisation of $(AC)_n$ microsatellite genetic markers from human chromosome 16. *Genomics* **13:** 402–408

Tsuda H and Hirohashi S (1995) Identification of multiple breast cancers of multicentric origin by histological observations and distribution of allele loss on chromosome 16q. *Cancer Res* **55:** 3395–3398

Tsuda H, Zhang W, Shimosato Y, Yokota J, Terada M, Sugimura T, Miyamura T and Hirohashi S (1990) Allele loss on chromosome 16 associated with progression of human hepatocellular carcinoma. *Proc Natl Acad Sci USA* **87:** 6791–6794

Tsuda H, Callen DF, Fukutomi T, Nakamura Y and Hirohashi S (1994) Allele loss on chromosome 16q24.2–qter occurs frequently in breast cancers irrespectively of differences in phenotype and extent of spread. *Cancer Res* **54:** 513–517

© Cancer Research Campaign 1997